# From Neuron‐Centric to Glia‐Centric: How Aging Glial Networks Drive Neurodegenerative Disease

**DOI:** 10.1111/jnc.70361

**Published:** 2026-01-27

**Authors:** Lívia de Sá Hayashide, Bruna Pessoa, Gustavo Dias, Bruno Pontes, Rafael Serafim Pinto, Luan Pereira Diniz

**Affiliations:** ^1^ Instituto de Ciências Biomédicas, Universidade Federal do Rio de Janeiro Rio de Janeiro Rio de Janeiro Brazil; ^2^ Centro Nacional de Biologia Estrutural e Bioimagem (CENABIO), Universidade Federal do Rio de Janeiro Rio de Janeiro Rio de Janeiro Brazil; ^3^ Instituto de Educação Médica (IDOMED) Rio de Janeiro Rio de Janeiro Brazil

**Keywords:** astrocytes, brain aging, glial senescence, neurodegeneration, senescence‐associated secretory phenotype, Senotherapy

## Abstract

The traditional neuron‐centric view of neurodegeneration is being replaced by a glial network–based framework. This shift recognizes that age‐related dysfunction in non‐neuronal cells critically shapes neuronal vulnerability and circuit resilience. Aging, the major risk factor for neurodegenerative diseases, is increasingly associated with the accumulation of senescent glial cells, particularly astrocytes, which emerge as early and active drivers of central nervous system decline. This review highlights astrocytic senescence as a key mechanism linking brain aging to neurodegeneration. Senescent astrocytes exhibit hallmark features including stable cell cycle arrest, mitochondrial dysfunction, and the acquisition of a senescence‐associated secretory phenotype (SASP), which disrupts synaptic integrity, impairs proteostasis, and sustains chronic neuroinflammation. These alterations often precede overt neuronal loss in disorders such as Alzheimer's and Parkinson's disease. We discuss core hallmarks and biomarkers of glial senescence, emphasizing integrative strategies combining functional assays and molecular markers. We further highlight circulating SASP‐related factors and extracellular vesicles as translational indicators of systemic senescence. Finally, we examine emerging senotherapeutic approaches aimed at restoring glial homeostasis, including senolytics, senomorphics, and CAR‐T–based immunotherapies. Targeting glial senescence and interglial communication therefore represents a promising, though complex, paradigm‐shifting avenue for delaying brain aging and mitigating neurodegenerative progression.

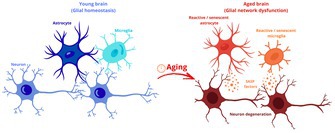

## Graphical abstract

During aging, progressive alterations in glial networks reshape intercellular communication within the central nervous system. In the young brain, astrocytes and microglia actively regulate metabolic, immune, and synaptic processes that sustain neural circuit stability. With advancing age, these cells acquire reactive and senescent phenotypes, accompanied by dysregulated signaling and the release of senescence‐associated secretory phenotype (SASP) factors. This glial network dysfunction amplifies neuroinflammation, disrupts synaptic organization, and reduces neuronal resilience, ultimately driving region‐specific neuronal degeneration in the aged brain.

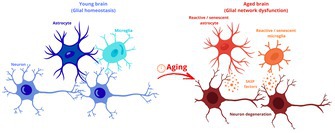

**Abbreviations**
AD: Alzheimer's disease
Aβ: beta‐amyloid
CAR‐T: chimeric antigen receptor T cells
CDK: cyclin‐dependent kinase
CNS: central nervous system
EVs: extracellular vesicles
GFAP: glial fibrillary acidic protein
IL: interleukin
MMP: matrix metalloproteinase
mTOR: mechanistic target of rapamycin
NF‐κB: nuclear factor kappa B
NKG2D: natural killer group 2D receptor
PD: Parkinson's disease
ROS: reactive oxygen species
SASP: senescence‐associated secretory phenotype
SA‐β‐gal: senescence‐associated beta‐galactosidase
TGF‐β1: transforming growth factor beta 1
TIFs: telomere dysfunction‐induced foci
TNT: tunneling nanotubes
uPAR: urokinase‐type plasminogen activator receptor
α‐syn: α‐synuclein

## 1 Introduction

For decades, glial cells were largely framed as passive supporters of neuronal structure and survival (Miguel‐Hidalgo 2023; York et al. 2018). This view has shifted profoundly, as experimental and omics‐driven neuroscience established that glia actively shape synaptic function, metabolic homeostasis, immune surveillance, and tissue repair across the lifespan (Fan and Agid 2018). In neurodegenerative disorders, the historical neuron‐centric focus captured the most visible endpoint, neuronal dysfunction and death, but increasingly failed to explain why vulnerability emerges in specific circuits, why pathology propagates over time, and why inflammatory and metabolic signatures precede overt neuronal loss.

Aging provides a unifying context for these questions. With age, glial cells undergo functional remodeling that can be adaptive at first but progressively becomes maladaptive, including impaired proteostasis, lysosomal and mitochondrial dysfunction, altered phagocytosis, and chronic inflammatory signaling (Pan et al. 2020). Accumulating evidence supports the emergence of senescence‐like glial states in the aged brain, characterized by stable cell cycle arrest programs, a senescence‐associated secretory phenotype (SASP), and organelle dysfunction. These changes are not isolated. Glial populations engage in continuous bidirectional communication, and dysfunction in one glial compartment can reprogram the other, ultimately reshaping the neuronal microenvironment.

In this review, we examine the conceptual transition from neuron‐centric frameworks to glial network–based models of neurodegeneration, with a particular focus on astrocytes as early sensors and active modulators of brain aging. We discuss key hallmarks of astrocytic senescence, the contribution of inflammaging and circulating senescence‐associated biomarkers, and emerging therapeutic strategies targeting senescent cells in the aging brain, including senotherapies and CAR‐T–based approaches.

## 2 From Neuron‐Centric Views to Glial Networks in Neurodegeneration

**FIGURE 1:**
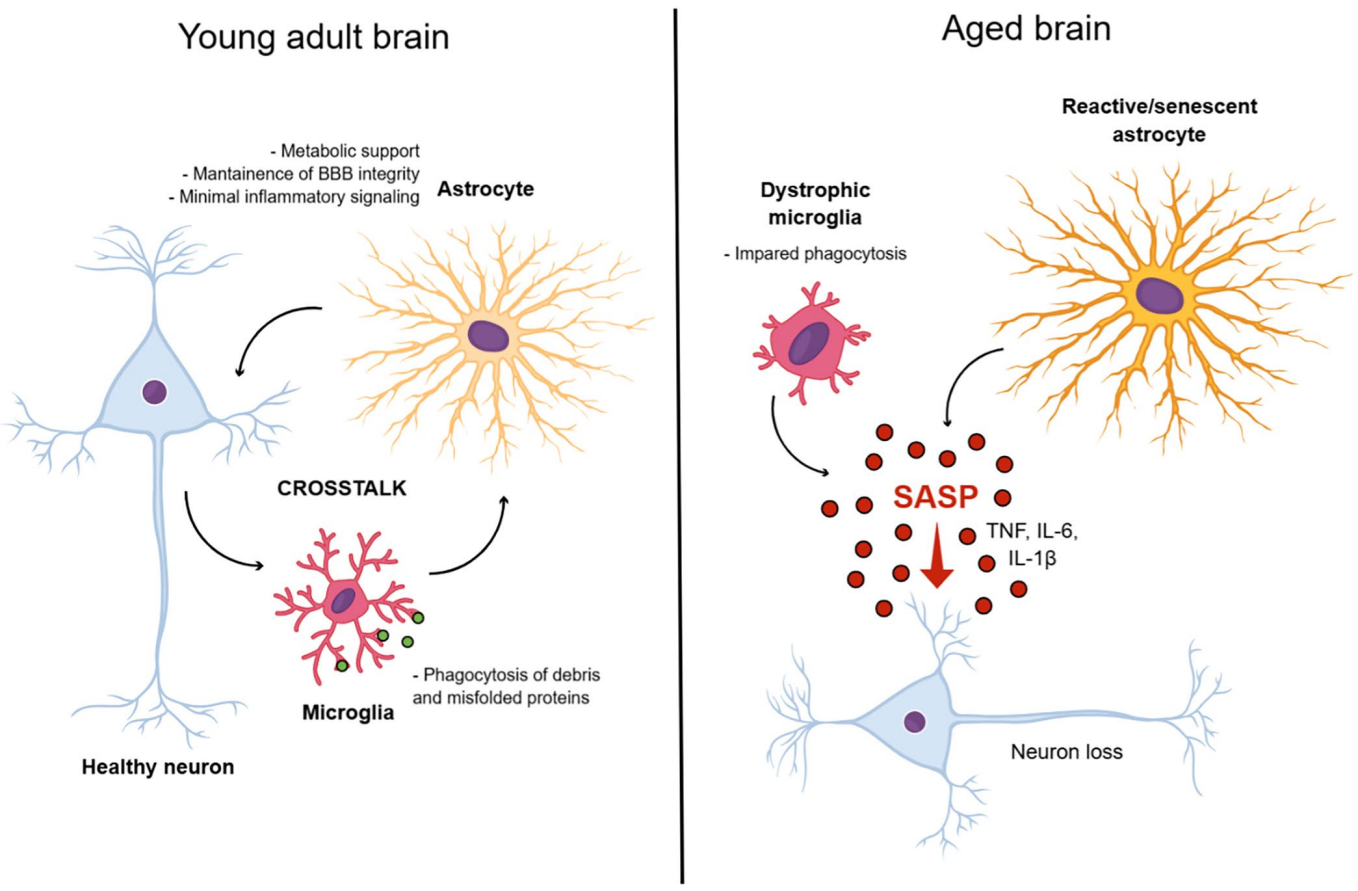
Glial crosstalk in the young adult brain versus the aged brain. Schematic comparison of cellular interactions in the young adult brain and during brain aging. In the young adult brain, astrocytes support neuronal homeostasis through metabolic coupling, maintenance of blood–brain‐barrier integrity, and minimal inflammatory signaling, while microglia efficiently phagocytose debris and misfolded proteins. This balanced astrocyte–microglia–neuron crosstalk sustains neuronal health. In the aged brain, microglia acquire a dystrophic phenotype with impaired phagocytic capacity, and astrocytes become reactive or senescent. Senescent astrocytes release a senescence‐associated secretory phenotype enriched in pro‐inflammatory mediators such as TNF, IL‐6, and IL‐1β, amplifying neuroinflammation and disrupting glial–neuronal communication. These changes contribute to neuronal dysfunction and progressive neuron loss during aging.

Aging is the primary risk factor for most neurodegenerative diseases and profoundly reshapes the cellular and molecular landscape of the central nervous system (CNS) (Hou et al. 2019). These changes emerge long before overt neuronal loss and clinical symptoms, indicating that neurodegeneration develops on a background of gradual cellular dysfunction. Increasing evidence indicates that age‐associated remodeling of glial cells plays a central role in this process, as glia integrate metabolic, inflammatory, and synaptic signals that ultimately determine neuronal vulnerability and circuit resilience (Figure 1) (Picca et al. 2022).

Glial cells undergo profound alterations during aging and in neurodegenerative conditions, acting not only as secondary responders to neuronal injury but also as active contributors to disease initiation and progression. Notably, astrocytic dysfunction can precede overt neuronal damage. In an oligomeric beta‐amyloid (Aβ)–induced toxicity model, astrocytes exhibit marked morphological atrophy and a loss of synaptogenic capacity. Importantly, restoration of astrocytic morphology and synaptogenic function through transforming growth factor beta 1 (TGF‐β1) treatment was sufficient to rescue Aβ‐induced synaptic loss and memory impairment (Diniz et al. 2017). These findings support the concept that astrocytic dysfunction is not merely a downstream consequence of neuronal degeneration but may represent an early and causative event in Alzheimer's disease (AD)–related neurodegenerative processes (Diniz et al. 2017). Beyond cell‐autonomous alterations, glial cells engage in constant intercellular communication through multiple mechanisms that will be better explored in the following sections of this review, influencing one another's responses. In this context, understanding glial network dynamics has become essential for elucidating the pathophysiological mechanisms underlying neurodegenerative diseases.

Evidence indicates a progressive accumulation of senescent glial cells in the aging brain, particularly astrocytes, which emerge as early and active contributors to age‐associated neural dysfunction (Sikora et al. 2021; Araujo et al. 2024). Upon entering a senescent state, astrocytes exhibit classical hallmarks of senescence, including stable cell cycle arrest, profound lysosomal and mitochondrial dysfunction, and the acquisition of SASP (Cohen and Torres 2019). This profile is enriched in pro‐inflammatory cytokines, chemokines, and matrix‐remodeling enzymes that disrupt synaptic homeostasis, impair metabolic and trophic support, and alter neuron–glia communication. Importantly, the astrocyte‐driven changes often precede overt neuronal loss, positioning astrocytic senescence as a potential initiating event in brain aging rather than a mere consequence of neurodegeneration (Salminen et al. 2011; Chinta et al. 2018).

By reshaping the local microenvironment and influencing neighboring microglia and neurons, senescent astrocytes may therefore set the stage for the onset and progressive amplification of neurodegenerative processes.

In Parkinson's disease (PD), microglial cells are found in high abundance within classically affected regions such as the substantia nigra pars compacta and the ventral tegmental area (Shaerzadeh et al. 2020). These microglia closely associate with dopaminergic neurons, acting as sentinels that provide trophic and immune support under physiological conditions. Under normal conditions, they play a key role in clearing extracellular α‐synuclein (α‐syn) aggregates through autophagy–lysosome–dependent degradation (Choi et al. 2020). However, in the aged mouse brain, microglia acquire a senescent phenotype characterized by impaired phagocytic capacity and dysfunctional lysosomal/autophagic machinery leading to an intracellular accumulation of misfolded α‐syn. This functional decline compromises α‐syn clearance and contributes to its extracellular accumulation in the microglia of aged mice, thereby exacerbating dopaminergic neurodegeneration (Choi et al. 2020; Hong et al. 2024).

Similar mechanisms have been described in AD, where specific subpopulations known as disease‐associated microglia exhibit enhanced capacity to phagocytose β‐amyloid plaques (Keren‐Shaul et al. 2017). Nevertheless, during aging and chronic Aβ exposure, these cells can also undergo functional senescence, resulting in reduced Aβ clearance and the secretion of a proinflammatory SASP that perpetuates neuroinflammation (Samuel Olajide et al. 2024).

In this context, astrocytes and microglia exhibit coordinated and complementary roles in regulating both protein clearance and apoptotic cell removal (Damisah et al. 2020). Efficient clearance of misfolded and aggregated proteins, such as α‐syn and Aβ, critically depends on the dynamic crosstalk between astrocytes and microglia (Rostami et al. 2021). Astrocytes play a dual role—they engulf aggregates and transfer them to microglia via secretory pathways, including extracellular vesicle–mediated mechanisms. In PD brain, astrocytes are frequently observed in proximity to infiltrating T cells and can act as antigen‐presenting cells. After astrocytic secretion of these protein‐containing cargos, microglia execute the terminal clearance through phagocytosis and subsequent degradation (Rostami et al. 2020).

Astrocytes can exert potent neuroprotective effects through the secretion of TGF‐β1. In PD models, TGF‐β1 signaling promoted synaptogenesis, restored the expression and function of astrocytic glutamate transporters, and enhanced neuronal resilience to neurotoxic insults. These findings highlight TGF‐β1–mediated astrocyte–neuron communication as a key mechanism for preserving synaptic integrity and excitatory balance in neurodegenerative conditions (Diniz et al. 2017, 2019).

Astrocytic function also displays marked regional heterogeneity. Astrocytes residing in the ventral midbrain are particularly important, as they exhibit neuroprotective and therapeutic properties (Yang et al. 2022). These cells help reduce oxidative stress and mitochondrial dysfunction, in addition to promoting the disaggregation of α‐syn. Furthermore, transplantation of ventral midbrain astrocytes into PD mouse models has been shown to improve disease pathology and protect dopaminergic neurons from degeneration (Song et al. 2018).

This regional and subtype specificity was further demonstrated by Sadick et al. (2022), in which astrocytes and oligodendrocytes were shown to alter their gene expression profiles in the brains of individuals with AD. In neurodegenerative disorders, glial cells appear to undergo a series of phenotypic and functional changes, suggesting that their responses are both complex and heterogeneous. These alterations vary according to cellular location and subtype, which may help explain why certain brain regions are more susceptible to neurodegeneration than others (St‐Pierre et al. 2022).

A defining feature of brain aging and neurodegenerative disease is the selective vulnerability of specific brain regions, which cannot be explained solely by neuronal intrinsic properties. Increasing evidence indicates that regional heterogeneity of glial cells contributes substantially to this selective susceptibility (Cragnolini et al. 2020). Astrocytes and microglia display region‐specific metabolic profiles, inflammatory thresholds, mitochondrial dynamics, and proteostatic capacity, which may render certain circuits, such as the hippocampus, entorhinal cortex, and substantia nigra, particularly sensitive to aging‐associated stress (Tan et al. 2020; Sun et al. 2022). Omics‐based studies and spatial transcriptomics support the existence of distinct glial states across brain regions (Tan et al. 2020; Schroeder et al. 2025), suggesting that differential engagement of senescence programs may underlie regionally restricted neurodegeneration. Aging induces region‐specific transcriptional reprogramming in astrocytes, characterized by the selective activation of synapse‐elimination and stress‐response pathways while core homeostatic and neurotransmission‐supporting functions remain largely preserved. This astrocytic shift generates a microenvironment permissive to synaptic loss and neuronal dysfunction, providing a mechanistic link between glial aging, circuit vulnerability, and cognitive decline (Boisvert et al. 2018). In parallel, human microglia undergo a profound transcriptional and functional reprogramming, giving rise to an age‐associated phenotype that is molecularly distinct from both homeostatic and acutely reactive states and is tightly associated with genetic risk factors and pathogenic pathways underlying neurodegenerative diseases (Olah et al. 2018).

In aging and neurodegenerative diseases, astrocytes lose their neuroprotective capacity and become activated into a reactive state (Clarke et al. 2018). Reactive astrocytes are defined as astrocytes that undergo context‐dependent molecular, morphological, and functional remodeling in response to injury, inflammation, or neurodegenerative stimuli. This state is characterized by changes in gene expression, cellular hypertrophy or atrophy, altered metabolic and homeostatic functions, and the acquisition of inflammatory or neuroprotective phenotypes (Wang et al. 2023; Jiwaji et al. 2022). Importantly, astrocyte reactivity is largely driven by microglia‐derived signals, positioning interglial communication as a key determinant of whether reactive astrocytes exert protective or deleterious effects during aging and neurodegenerative diseases (Liddelow et al. 2017).

It has been shown that abnormal astrocyte reactivity can determine Aβ‐triggered tau pathology (Bellaver et al. 2023). Astrocytes are capable of accumulating tau fibrils, which may in turn promote propagation of tau aggregates to neighboring cells, induce neuroinflammation, and cause neuronal damage (Eltom et al. 2024). Moreover, oligodendrocytes also contribute directly to amyloid plaque formation, further emphasizing the involvement of multiple glial cell types in the pathological landscape of neurodegenerative diseases (Sasmita et al. 2024). Overall, these findings indicate that neurodegeneration arises from progressive dysfunction of interconnected glial networks, rather than neuron‐autonomous processes alone (Boisvert et al. 2018; Zhang et al. 2022).

An important unresolved issue concerns the temporal relationship between astrocytic senescence, microglial activation, and neuronal dysfunction. Although multiple experimental models suggest that astrocytic alterations can precede synaptic failure and neuronal loss, particularly in early stages of aging and amyloid or tau pathology, available evidence does not support a single linear sequence of events (Canals et al. 2023; Briel et al. 2021; Bellaver et al. 2023). Instead, astrocytic senescence, microglial reactivity or senescence, and neuronal stress appear to emerge in partially overlapping and context‐dependent temporal windows. Importantly, causality is more robustly supported in experimental systems, whereas human postmortem studies and circulating biomarker analyses largely capture correlational associations. This temporal complexity is essential, as it suggests that glial senescence may act both as an initiating factor and as an amplifier of neurodegenerative cascades, depending on disease stage, brain region, and systemic inflammatory context.

## 3 Astrocytes as Early Sensors of Brain Aging and Neurodegeneration

Against this background, aging represents the key biological context in which these dysfunctions unfold. Rather than reflecting uniform cell death, brain aging is characterized by subtle but cumulative phenotypic and functional changes across distinct glial populations (Bernal and Peterson 2011; Li et al. 2023; Hoshino et al. 2024; Edler et al. 2023).

During physiological aging, the total number of neurons and overall synaptic density remain largely preserved, whereas synaptic architecture, particularly synapse size and structural organization, is altered (Freeman et al. 2008), often preceding overt neuronal death. This is especially relevant, given that glial cells actively release soluble factors and vesicular cargos that may serve as early biomarkers of senescence (Li et al. 2021).

During aging, both the number and phenotype of glial cells are altered, and all major glial populations undergo functional changes. The number of oligodendrocytes, crucial for rapid neural transmission through myelin sheath and metabolic support to neurons, declines with aging which explains a decreased myelination and cognitive decline in the elderly people (Dimovasili et al. 2023). In contrast, the number of microglia remains stable in humans throughout aging (Menassa et al. 2022). However, aging leads to microglial dysfunction, contributing to neurodegeneration. This aged microglia enter a state called “priming”, characterized by increased expression of major histocompatibility complex class II and inflammatory markers, but with reduced phagocytic capacity and the accumulation of dysfunctional microglia in the hippocampus, substantia nigra and cortex associated with the chronic release of pro‐inflammatory cytokines and neuronal damage (Peters 2009; Bartzokis 2004; Li et al. 2023; Qu and Li 2020).

Similarly to microglia, astrocytes do not appear to undergo cell death during aging; instead, they exhibit pronounced phenotypic and functional remodeling. Their morphology, functionality, and expression profile change significantly. In regions such as the hippocampus and prefrontal cortex, astrocytes display hypertrophy, increased glial fibrillary acidic protein (GFAP) expression, and metabolic dysfunction, features that may overlap with reactivity or senescence (Pelvig et al. 2008). Our group showed that aged mice and elderly humans display deformation of astrocytic nuclei in the hippocampus accompanied by reduced Lamin B1 levels. As Lamin B1 loss is a hallmark of cellular senescence, these alterations indicate profound nuclear and transcriptional dysregulation in astrocytes, with potential consequences for synaptic support, glial homeostasis, and cognitive function during aging (Matias et al. 2022).

Dysregulated astrocytic reactivity has been implicated in the pathophysiology of several neurodegenerative and neuroinflammatory conditions, including AD, PD, and multiple sclerosis, highlighting their critical role in neural circuit stability and repair mechanisms (Sofroniew and Vinters 2010; Liddelow et al. 2017; Colombo and Farina 2016). In this context, accumulating evidence indicates that astrocytic dysfunction in neurodegenerative and neuroinflammatory diseases is closely linked to the acquisition of a senescent phenotype.

Cellular senescence is a complex and multifaceted process that affects several cell types, including glial cells such as astrocytes. Senescent astrocytes accumulate with aging and in neurodegenerative diseases such as AD (Bussian et al. 2018), PD (Xia et al. 2020), and amyotrophic lateral sclerosis (Vazquez‐Villaseñor et al. 2020). Unlike fibroblasts or epithelial cells, senescent astrocytes present a partially distinct senescence profile, requiring a combination of markers for their robust and specific detection. Cellular senescence in astrocytes is implicated in neurodegenerative diseases, brain aging, and CNS injuries. Senescent astrocytes exhibit distinct morphological, functional, and molecular alterations (Matias et al. 2022; Kim et al. 2024; Russo et al. 2025).

Senescent astrocytes may exacerbate neuronal dysfunction by losing supportive functions and acquiring deleterious pro‐inflammatory features, thereby further compromising CNS homeostasis (Chinta et al. 2015; Crowe et al. 2016). Our group demonstrated that senescent astrocytes exhibit upregulation of glutamate transporters and glutamine synthetase in vitro and in the mouse and human hippocampus. This finding highlights a dual role of astrocytic senescence, in which senescent astrocytes may simultaneously acquire deleterious pro‐inflammatory features while preserving or compensatory enhancing specific homeostatic functions related to glutamate metabolism (Matias et al. 2023).

Senescent astrocytes also display marked alterations in mitochondrial dynamics, characterized by enhanced mitochondrial fission (Araujo et al. 2024) and the accumulation of damaged mitochondria (Diniz et al. 2024), likely due to impaired mitophagy. This mitochondrial dysfunction contributes to increased oxidative stress and reduced cellular resilience. Notably, activation of mitophagy through mechanistic target of rapamycin (mTOR) pathway modulation has been shown to reverse key markers of astrocytic senescence and to reduce astrocyte susceptibility to mitochondrial stress. These findings indicate that defective mitochondrial quality control is a central mechanism sustaining astrocytic senescence and highlight mitophagy as a potential therapeutic target to restore astrocyte function during brain aging and neurodegeneration.

Reactive astrocytes share several features with senescent cells, and senescent astrocytes increase with age, potentially reshaping the CNS microenvironment and contributing to age‐associated neurological pathologies (Cohen and Torres 2019). Astrocytes are particularly affected in disorders associated with age‐related cognitive decline, including AD and PD (Liddelow et al. 2017; Diniz et al. 2017).

During aging and neurodegenerative diseases, astrocytes undergo morphological and functional changes that can contribute to cognitive decline and pathology progression (Rodríguez‐Arellano et al. 2016). These changes include alterations in antioxidant protection (Tsesmelis et al. 2023), neurovascular coupling (Tarantini et al. 2017), and glutamate clearance (Barreto et al. 2011). Astrocytic autophagy, a key cellular process for protein and organelle degradation, is implicated in inflammation, oxidative stress, and aging (Wang and Xu 2020). These alterations lead to reduced support for neurons and increased neuroinflammation (Palmer and Ousman 2018). In AD, astrocytes undergo early degeneration and atrophy, potentially contributing to cognitive deficits, while later stages are characterized by reactive astrogliosis associated with amyloid plaques (Rodríguez‐Arellano et al. 2016).

Senescent astrocytes play a significant role in AD. These cells exhibit decreased normal physiological functions and increased secretion of SASP factors, contributing to Aβ accumulation, tau hyperphosphorylation, neuroinflammation, and synaptic dysfunction (Han et al. 2020; Kim et al. 2024; Meldolesi 2023). The accumulation of senescent astrocytes contributes to cognitive decline and the development of various neurodegenerative disorders. Recent research has focused on targeting astrocyte senescence as a potential therapeutic approach for AD, with senolytic compounds being tested in clinical trials (Tuzer and Torres 2022). Additionally, treating senescent astrocytes with chemicals targeting reactive oxygen species (ROS) or mitochondrial function may reverse senescence phenotypes (Kim et al. 2024).

During aging and AD, astrocytes progressively acquire senescence‐associated features that reshape their morphology and function. Studies have shown an increase in senescent astrocytes expressing p16INK4a and matrix metalloproteinase 1 (MMP‐1) in aged and Alzheimer's brains (Bhat et al. 2012). Age‐dependent remodeling of astrocytes varies by brain region, with hypertrophy observed in hippocampal areas and atrophy in the entorhinal cortex (Rodríguez et al. 2014). GFAP, a marker of astrocyte reactivity, consistently increases with age in mouse models, while other markers like glutamine synthetase and S100β show variable changes (Rodríguez et al. 2024). Reactive and senescent astrocytes exhibit distinct phenotypes, both contributing to neuroinflammation and loss of homeostatic functions in neurodegenerative diseases (Lazic et al. 2022). Despite these overlaps, it remains unclear whether senescent astrocytes share the same molecular and functional profile as reactive astrocytes, and whether these states represent distinct or partially overlapping phenotypes.

A study demonstrated an association between astrocyte reactivity and synaptic dysfunction in elderly humans. This evidence supports the notion that astrocytes may play a crucial role in synaptic maintenance, and that their dysfunction can lead to synaptic loss (Rohden et al. 2025). Consistent with these observations, recent evidence indicates that across the AD spectrum, Aβ deposition is associated with astrocyte reactivity only in the context of microglial activation, and that this microglia–astrocyte axis contributes to cognitive decline by promoting tau phosphorylation and aggregation (Ferrari‐Souza et al. 2025).

## 4 Microglial Senescence in Brain Aging

While astrocytic senescence has received increasing attention as a driver of brain aging, accumulating evidence indicates that microglia also acquire senescence‐like features during aging and neurodegenerative disease (Flanary et al. 2007). Senescent microglia are characterized by stable cell cycle arrest, altered chromatin organization, impaired phagocytic capacity, and a sustained pro‐inflammatory secretory profile that partially overlaps with the SASP (Li et al. 2023; Qu and Li 2020). Recent studies have identified increased expression of p16INK4a, accumulation of lipofuscin, lysosomal dysfunction, and metabolic rewiring as hallmarks of aged and senescent microglia, accompanied by reduced clearance of protein aggregates and cellular debris (Rim et al. 2024; Wei et al. 2023).

Single‐cell and spatial transcriptomic analyses further revealed that microglial senescence signatures are not uniformly distributed across the brain, but instead emerge preferentially in vulnerable regions, suggesting region‐specific susceptibility to senescence programs (Olah et al. 2018; Boche and Gordon 2022). Importantly, senescent microglia exhibit impaired immunosurveillance and exaggerated inflammatory responses (Liu et al. 2025), which may synergize with astrocytic senescence to disrupt synaptic homeostasis and neuronal resilience.

Despite these advances, distinguishing microglial senescence from chronic activation or priming remains challenging, as these states share partially overlapping transcriptional and functional features. Importantly, several molecular stimuli classically associated with microglial reactivity, including aggregated proteins such as tau and Aβ, have also been implicated in the induction of senescence‐associated programs under conditions of chronic or sustained exposure (Karabag et al. 2023; An et al. 2022). These observations raise the possibility that microglial senescence may, in some contexts, emerge downstream of prolonged or unresolved reactive states, rather than constituting a fully discrete phenotype. However, current evidence remains insufficient to establish a direct or universal trajectory from reactivity to senescence. Further integrated functional, molecular, and temporal studies will be required to determine whether and when reactive microglia transition into senescence and to clarify the contribution of senescent microglia to neurodegenerative disease progression.

As a result, accurately identifying senescent glial cells in tissues requires robust and well‐validated biomarkers. In the following sections, we discuss the principal markers of cellular senescence and the methodologies currently used for their detection.

## 5 Hallmarks and Biomarkers of Glial Senescence

**FIGURE 2:**
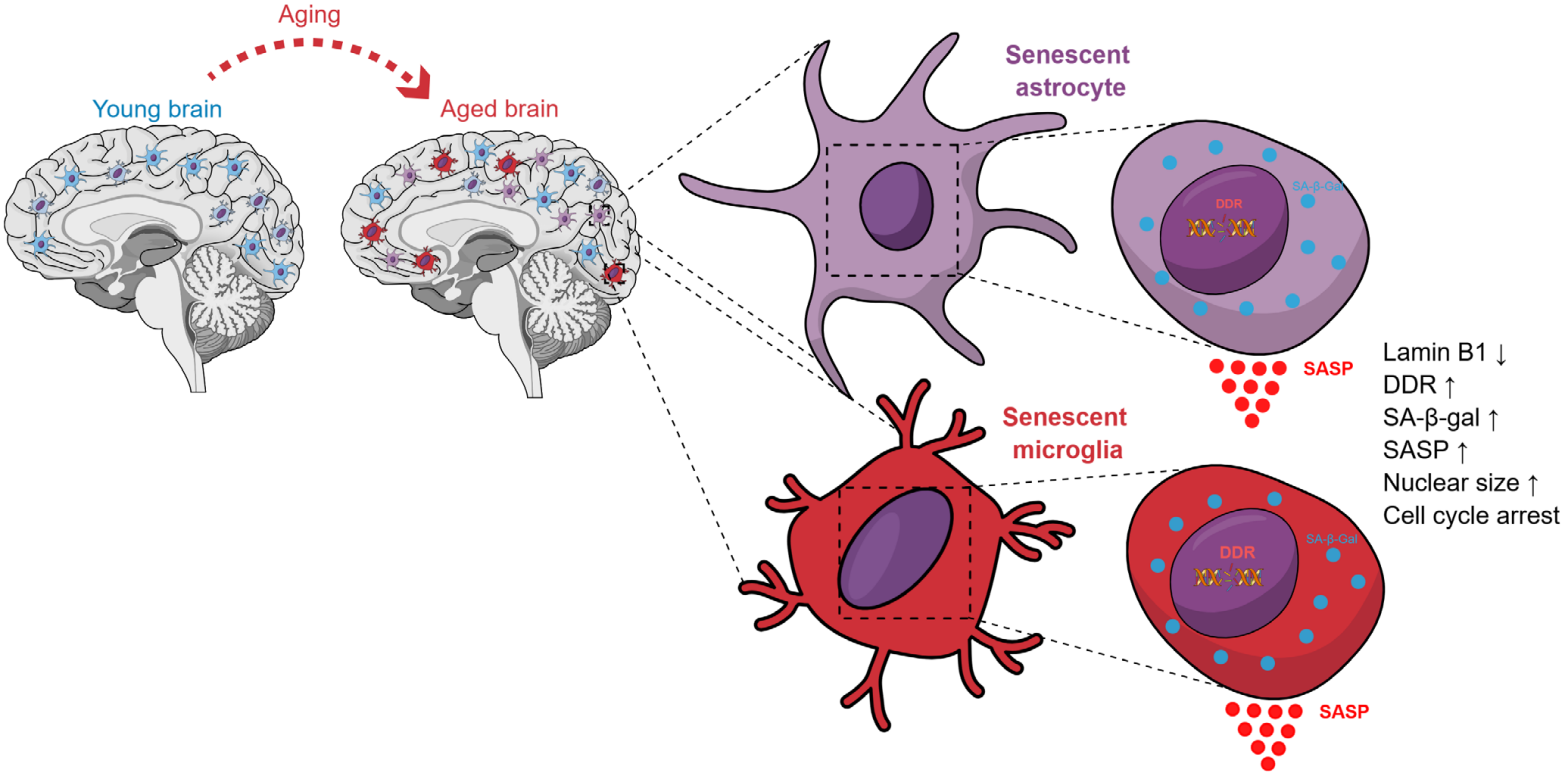
Glial senescence in the aging brain. Schematic representation of the transition from the young brain to the aged brain, highlighting the accumulation of senescent glia during aging. In the aged brain, astrocytes and microglia acquire a senescent phenotype characterized by reduced Lamin B1 levels, sustained activation of the DNA damage response, increased senescence‐associated β‐galactosidase activity, nuclear enlargement, and stable cell cycle arrest. Senescent astrocytes also develop a senescence‐associated secretory phenotype, marked by the release of pro‐inflammatory and neuroactive factors that can disrupt tissue homeostasis and contribute to age‐related brain dysfunction.

Given the phenotypic heterogeneity of senescent glia and the substantial overlap between senescence and reactive states, the identification of reliable biomarkers requires integrative, well‐characterized approaches (Figure 2). Recently, we described a robust in vitro model of astrocyte senescence induced by doxorubicin in both human primary and murine astrocytes, characterized by increased expression of senescence markers p21 and senescence‐associated beta‐galactosidase (SA‐β‐gal), activation of the DNA damage response (γ‐H2AX and 53BP1), nuclear enlargement, cell cycle arrest, and a pronounced pro‐inflammatory SASP marked by elevated MMP3, IL‐6, and IL‐1β. This human‐relevant model provides a valuable platform to investigate the molecular mechanisms linking astrocyte senescence to age‐related neuroinflammation and neurodegeneration, complementing and extending insights from traditional murine systems (Marques et al. 2025).

Among classical hallmarks of cellular senescence, SA‐β‐Gal remains one of the most widely used and experimentally accessible markers. β‐galactosidase is a hydrolase belonging to the glycosidase family, whose primary function is to catalyze the hydrolysis of β‐galactose residues into monosaccharides. This enzyme is conserved across species, from bacteria to mammals, where it plays essential roles in cellular metabolism and regulation (Kurz et al. 2000).

Dimri et al. were the first to identify pH 6.0 β‐galactosidase as a biomarker of cellular senescence but also established a histochemical detection method using the chromogenic substrate X‐Gal (5‐bromo‐4‐chloro‐3‐indolyl β‐D‐galactopyranoside). This assay exploits enzymatic activity at suboptimal lysosomal pH (6.0 versus the canonical ~4.0), where X‐Gal cleavage generates an insoluble indigo precipitate, allowing direct visualization of senescent cells in culture and tissue samples. Since its introduction, this method has become the most widely used approach for detecting senescent cells in vitro and in vivo, particularly in studies of aging, cancer, and degenerative diseases, as it enables discrimination between senescent and quiescent cells within heterogeneous populations (Dimri et al. 1995). However, despite its broad use, this approach is largely qualitative and not compatible with paraffin‐embedded tissues, limiting its applicability in clinical samples.

For quantitative analyses in living cells, modern techniques have been developed. One widely used fluorescent probe is C12FDG (5‐dodecanoylfluorescein di‐β‐D‐galactopyranoside), which is cleaved by SA‐β‐Gal, releasing fluorescein that can be detected by flow cytometry or fluorescence microscopy. However, efficient detection requires pre‐incubation with bafilomycin A1, a drug that alkalinizes lysosomes, allowing enzymatic activity to occur efficiently at the desired pH (Debacq‐Chainiaux et al. 2009).

Highly sensitive fluorogenic probes such as SPiDER‐β‐Gal have been developed. These probes bind to intracellular components after enzymatic cleavage, enabling high‐resolution, real‐time imaging of SA‐β‐Gal activity in living cells. Compared to X‐Gal, SPiDER‐β‐Gal offers greater sensitivity, improved specificity, and broader applicability in clinical and translational settings (Itahana et al. 2007). The SPiDER‐β‐Gal platform has been extensively applied in clinical oncology and senolytic drug screening, owing to its high sensitivity and low background signal. Fluorescence is emitted only upon enzymatic cleavage by SA‐β‐Gal, allowing real‐time in vivo detection of β‐galactosidase activity. This method has been successfully used in glioblastoma studies (Nakamura et al. 2017; Kubo et al. 2021) and has more recently been employed in anti‐aging therapy research (Zonari et al. 2023; Cai et al. 2020).

Significant efforts are currently being made to optimize SA‐β‐Gal detection methods and translate them into clinical applications. These advances are particularly relevant for aging research, as they enable non‐invasive or minimally invasive assessment of senescence burden. Notably, emerging approaches aim to detect SA‐β‐Gal activity using positron emission tomography (PET), representing a highly promising strategy for studying brain aging due to its non‐invasiveness and feasibility in human subjects (Xiang et al. 2024).

In oncology, SA‐β‐Gal detection is already employed as a prognostic marker, helping to assess disease progression (Wagner et al. 2015; Choi et al. 2000). Its established use in cancer research highlights its potential relevance for studying cellular senescence. As a biomarker, SA‐β‐Gal could provide crucial insights into the earliest signs of aging, serving as a valuable tool to address fundamental questions that remain open, such as: When does aging actually begin? and can we intervene to slow it down?

Although not exclusive to senescence, cells with high lysosomal activity may also stain positively. SA‐β‐Gal is considered a robust and easy‐to‐apply marker and its detection is considered a robust indicator (Itahana et al. 2007).

It is therefore important to highlight that SA‐β‐Gal alone, although a classic and useful marker, is not sufficient to confirm the senescent phenotype. Therefore, its association with other molecular and morphological markers is highly recommended. Beyond functional markers such as senescence‐associated β‐galactosidase, a defining feature of cellular senescence is the establishment of a stable and often irreversible cell cycle arrest (Kumari and Jat 2021). The regulation of senescence‐associated cell cycle arrest is complex and involves multiple proteins and signaling pathways. p53 is considered one of the most important transcription factors controlling the cell cycle. It regulates hundreds of genes and is found to be inactivated in more than 50% of cancer cases. Its activation is triggered by DNA damage, oncogene activation, oxidative stress, and other stimuli. The main mechanism of p53‐mediated action occurs through the induction of p21, which inhibits cyclin‐dependent kinases (CDKs), leading to cell cycle arrest in G1 or G2 and promoting apoptosis or cellular senescence (el‐Deiry et al. 1993).

In parallel, p16INK4a acts largely independently of p53 and typically induces a permanent cell cycle blockade (Jacobs and de Lange 2004), further reinforcing the senescent state. Consistent with this framework, senescent cells frequently exhibit elevated expression of p16INK4a and p21CIP1, as well as p53, reflecting sustained activation of DNA damage checkpoints and the enforcement of long‐term cell cycle arrest (Sharpless and Sherr 2015).

p16INK4a is one of the main markers of cellular senescence (Rayess et al. 2012). Its expression has been associated with a decline in neurogenesis in the brains of aged mice (Molofsky et al. 2006). Both gain‐ and loss‐of‐function studies have shown that its overexpression leads to a premature reduction in progenitor cell proliferation, mimicking an aged phenotype. Conversely, its deletion preserves the proliferative capacity of neural progenitors. These findings indicate that p16INK4a plays a causal role in the decline of neurogenesis observed during aging. Human studies further support a link between p16INK4a upregulation and neurodegeneration. Neurons from AD patients display elevated p16 levels compared to healthy controls, with increased CDKN2A mRNA expression in the prefrontal cortex, but not in white matter regions enriched in glial cells (Herdy et al. 2022). These findings suggest that the upregulation of p16 in neurons may be specifically associated with the neurodegenerative processes, rather than reflecting a generalized glial response.

Another important molecular hallmark of cellular senescence is the accumulation of persistent DNA damage foci, particularly those that localize to telomeric regions, known as telomere dysfunction‐induced foci (TIFs). Telomeres are especially vulnerable to replicative stress and oxidative damage, and their dysfunction triggers a sustained DNA damage response that cannot be efficiently resolved, thereby promoting stable cell cycle arrest (Hewitt et al. 2012). TIFs represent sites where canonical DNA damage markers, such as phosphorylated histone H2AX (γH2AX), colocalize with telomeric DNA, reflecting chronic activation of DNA damage signaling at chromosome ends. The detection of TIFs is typically achieved by combining immunofluorescence for γH2AX or other DNA damage response proteins with fluorescence in situ hybridization targeting telomeric sequences. The presence of TIFs is considered a robust indicator of senescence associated with telomere dysfunction, as these lesions persist independently of ongoing proliferation and reinforce the irreversible nature of the senescent state, particularly in aging and neurodegenerative contexts (d'Adda di Fagagna et al. 2003).

To strengthen the identification of the senescent phenotype, complementary approaches are commonly employed. Morphological assessment reveals that senescent cells typically display an enlarged and flattened morphology with increased cytoplasmic granularity. Additionally, the accumulation of lipofuscin, an autofluorescent pigment resulting from impaired lysosomal degradation and oxidative damage, constitutes a classical histological hallmark of cellular aging. Lipofuscin can be detected using probes such as SenTraGor (GL13), which is compatible with paraffin‐embedded tissues and has proven particularly valuable in translational and clinical studies (Evangelou et al. 2017). Furthermore, functional readouts of mitochondrial dysfunction and metabolic remodeling, including altered bioenergetic profiles, increased reactive oxygen species production, and shifts in cellular metabolism, are recognized as integral components of the senescent state. Together, these morphological, histochemical, and metabolic readouts enhance the robustness of senescence detection and provide critical insights into the functional consequences of cellular aging (Evangelou et al. 2017).

In addition to stable cell cycle arrest, SASP represents a central functional hallmark of senescent cells and a key mediator of their impact on tissue homeostasis (Gorgoulis et al. 2019). The SASP comprises a complex and dynamic repertoire of secreted factors, including pro‐inflammatory cytokines, chemokines, growth factors, proteases, and extracellular matrix–remodeling enzymes. Among the most frequently assessed components are interleukins such as IL‐6 and IL‐8, chemokines including MCP‐1 (CCL2), and matrix metalloproteinases such as MMP‐1 and MMP‐3. The expression of these factors can be detected using complementary approaches, including RT‐qPCR, ELISA, multiplex immunoassays, and transcriptomic analyses such as RNA‐seq and has been widely employed as an indicator of senescence in studies of aging and chronic inflammation (Coppé et al. 2008).

Beyond their role as biomarkers, SASP factors exert profound autocrine and paracrine effects, reshaping the local tissue microenvironment. Through sustained inflammatory signaling, SASP can reinforce senescence in an autocrine manner, induce senescence in neighboring cells via paracrine mechanisms, and disrupt tissue architecture and function. In the CNS, SASP components released by senescent glial cells are particularly relevant, as they can promote chronic neuroinflammation, impair synaptic function, alter neuronal excitability, and compromise neurovascular and metabolic support (Bussian et al. 2018; Limbad et al. 2020). Importantly, the composition and magnitude of the SASP are highly context‐dependent, varying according to cell type, senescence‐inducing stimulus, and tissue environment. This heterogeneity underscores the need for integrated SASP profiling rather than reliance on individual factors when assessing senescence in aging and neurodegenerative diseases.

## 6 Inflammaging and Circulating Biomarkers of Cellular Senescence

While cellular and tissue‐based markers provide critical insight into the mechanisms and spatial localization of senescence, their clinical applicability is inherently limited by restricted tissue accessibility. This constraint has driven growing interest in systemic biomarkers capable of capturing senescence‐associated processes in a minimally invasive manner. In this context, circulating components of the SASP and extracellular vesicles (EVs) have emerged as promising candidates, linking cellular senescence to systemic inflammation and organismal aging (Yin et al. 2021). Importantly, these circulating signatures offer a translational bridge between molecular senescence programs and their functional consequences in aging and neurodegenerative diseases.

Franceschi introduced the concept of “inflammaging” to describe the association between aging and a state of chronic, low‐grade systemic inflammation (Franceschi et al. 2018). This phenotype has been linked to a circulating SASP, which was first characterized in 2020 by Basisty et al., who demonstrated a correlation between the senescent cell secretome and circulating plasma proteins in humans (Basisty et al. 2020).

Inflammation has emerged as a ubiquitous and multifactorial phenomenon, and its chronic persistence increases cellular vulnerability, contributing to the functional decline of multiple organs, including the CNS. Moreover, it has been strongly associated with various age‐related diseases (Franceschi and Campisi 2014). This underscores the need to further explore and understand the pathways linking chronic inflammation, senescence, and aging.

Since these observations, researchers have focused on identifying plasma‐borne molecules that could serve as prognostic or diagnostic biomarkers of cellular senescence. In parallel, there has been growing recognition of the importance of investigating the molecular pathways underlying this process. More than 20 SASP‐related proteins have been identified in human plasma, including cytokines such as IL‐6 and IL‐1β, as well as matrix metalloproteinases, growth factors, and others (Basisty et al. 2020; Schafer et al. 2020). Each of these molecules is secreted by distinct cell types, and their expression levels appear to vary depending on the method of senescence induction, making the characterization of the human SASP profile extremely complex. Importantly, the circulating levels of these proteins show a direct correlation with age, reinforcing their potential as systemic biomarkers of aging and cellular senescence.

Human plasma also harbors distinct proteomic signatures of biological aging. Notably, large‐scale analyses revealed that 33.5% of age‐associated proteins correlate with mortality, while 45.3% are linked to multimorbidity, defined as the coexistence of multiple chronic diseases (Tanaka et al. 2020, 2018). These findings indicate that a substantial fraction of age‐modulated plasma proteins possess direct prognostic value, reinforcing that circulating proteomic signatures of aging represent clinically meaningful indicators of health status and aging trajectory rather than abstract biological correlates.

More recently, several studies have shown that EVs play a dual role contributing either to pathogenesis or exerting therapeutic effects through the transfer of bioactive molecules (Putri et al. 2025). EVs are released by senescent cells and have emerged as promising biomarkers for the early diagnosis of age‐related diseases, based on the analysis of their molecular cargo (Ryu et al. 2023).

Such exosomes derived from senescent cells exhibit distinct proteomic and lipidomic profiles compared with those released by non‐senescent cells, including an increased abundance of SASP‐related proteins and other molecules associated with inflammation and cellular stress (Patel et al. 2025). This suggests that senescence‐associated exosomes may serve as circulating indicators of cellular aging and provide valuable insights into the molecular mechanisms linking senescence, inflammation, and age‐related pathologies.

In patients with AD, EVs have been shown to contain microRNAs that may serve as potential prognostic biomarkers for the disease (Koh et al. 2021). Moreover, mitochondrial components have also been detected within these vesicles. Since mitochondrial dysfunction is a hallmark of AD, the presence of altered mitochondrial content and mitochondrial RNA within EVs further supports their relevance to disease pathology (Yao et al. 2021; Kim et al. 2020).

Beyond their potential as circulating biomarkers, SASP components play an active role in shaping the trajectory of brain aging and neurodegeneration. Chronic exposure to SASP‐derived inflammatory mediators contributes to a state of persistent low‐grade inflammation that increases cellular vulnerability, disrupts synaptic function, and impairs neurovascular and metabolic homeostasis (Garcia‐Dominguez 2025). In the CNS, SASP factors released by senescent glial cells can amplify neuroinflammatory signaling, promote paracrine senescence, and facilitate the propagation of neurodegenerative pathology (Acosta et al. 2013). Although extracellular vesicles represent a promising vehicle for capturing these signatures in a minimally invasive manner, current evidence must be interpreted with caution due to technical and methodological limitations, including variability in isolation protocols and cargo characterization. Validating SASP‐ and EV‐based biomarkers will require standardized methodologies and longitudinal human studies to define their specificity, temporal dynamics, and predictive value (Salvioli et al. 2023). Importantly, such integrated biomarker frameworks may not only improve diagnosis and disease stratification but also guide the rational application of emerging senotherapeutic strategies. The contribution of peripheral immune cells and immune–brain interfaces to these mechanisms remains insufficiently understood, warranting further investigation to clarify how systemic immune signaling intersects with glial network dysfunction during brain aging and neurodegeneration.

In addition to SASP factors and EVs, stressed and aging glial cells can also engage direct physical forms of intercellular communication. Among these, tunneling nanotubes (TNTs) have emerged as actin‐based membranous bridges that enable the direct transfer of organelles, proteins, and signaling molecules between connected cells (Palese et al. 2025). Although initially described in immune and cancer cells, considerably less explored and far less understood, increasing evidence indicates that astrocytes and microglia can form TNT‐like structures, particularly under conditions of metabolic stress, inflammation, and proteostatic overload (Xu et al. 2026; Palese et al. 2025). In the context of brain aging, such direct cytoplasmic continuity may represent an adaptive attempt to preserve network homeostasis, but it may also facilitate the spread of dysfunction across glial networks (Chakraborty et al. 2023; Dagar and Subramaniam 2023). Among the diverse cargos transported through TNTs, mitochondria have attracted particular attention due to their central role in cellular metabolism and stress adaptation (Dagar and Subramaniam 2023; Zhou et al. 2024). TNT‐mediated mitochondrial transfer has been reported as a mechanism by which stressed cells attempt to buffer metabolic failure by redistributing functional organelles (Palese et al. 2025). In glial networks, astrocytes have been shown to donate mitochondria to neurons or neighboring glia under injury and inflammatory conditions, promoting short‐term survival and functional recovery (Walters and Cox 2021). However, during aging and cellular senescence, impaired mitophagy and the accumulation of damaged mitochondria raise the possibility that TNTs may also serve as conduits for the propagation of mitochondrial dysfunction, oxidative stress, and inflammatory signals, thereby amplifying neurodegenerative processes (Palese et al. 2025). In this context, the next section discusses therapeutic approaches aimed at targeting senescent cells, including senolytic and senomorphic interventions, and their potential to modulate aging‐related neurodegenerative processes.

## 7 From Senescence Accumulation to Therapeutic Intervention in the Aging Brain

While circulating SASP factors and EVs provide valuable insights into the systemic burden of senescence and its association with aging and neurodegenerative diseases, they also highlight a more fundamental issue: the progressive accumulation of senescent cells within tissues. In the CNS, this accumulation reflects an imbalance between senescent cell generation and immune‐mediated clearance, which becomes increasingly compromised with age. As senescent cells persist and their secretory phenotype amplifies local and systemic dysfunction, targeting senescence itself has emerged as an attractive therapeutic strategy to modulate brain aging, preserve cognitive function, and attenuate neurodegenerative processes.

Although senescent cells can emerge at any stage of life, maintaining a balance between their production and clearance is essential (Demaria et al. 2014; Ovadya et al. 2018). When senescent cell production is low, the immune system can efficiently remove them; however, when their generation becomes excessive, accumulation occurs due to the immune system's inability to clear them effectively (Ovadya et al. 2018). Any impairment of immune function can therefore contribute to the accumulation of senescent cells, and during chronological aging, the progressive decline in immune surveillance results in a gradual buildup of senescent cells within tissues, promoting tissue dysfunction and age‐related decline (Ovadya et al. 2018; Baker et al. 2011).

Experimental evidence demonstrates that senescent cells are not merely markers of aging but active drivers of dysfunction. Transplantation of senescent cells into aged mice leads to a shortened lifespan (Xu et al. 2017). These findings provide direct causal support for targeting senescent cells therapeutically, motivating the development of interventions aimed at delaying CNS aging and mitigating SASP‐driven age‐related pathology. An important question is whether senescent cells should be eliminated in order to slow down or halt aging. Senescent cells can exert beneficial effects, and their complete elimination may lead to a range of detrimental consequences. Such questions touch on highly complex biological and ethical issues that are not easily addressed and must be examined from a multidisciplinary perspective.

In response to these challenges, a range of therapeutic strategies collectively termed senotherapies has emerged. Current approaches fall into two broad categories: senolytics, which selectively eliminate senescent cells (Xu et al. 2018), and senomorphics, which suppress or remodel the SASP without inducing cell death, thereby modulating the senescent phenotype (Laberge et al. 2015).

Among the most extensively studied senolytic candidates are quercetin, dasatinib, navitoclax, and fisetin. To date, none of these compounds have yet been approved for clinical use in the context of senescence or aging. Most of them are currently used in oncology, where their ability to induce apoptosis in specific cell populations was first characterized (Chang et al. 2016; Souers et al. 2013).

One of the first pieces of evidence suggesting that senescent cells could serve as a therapeutic target came from a study in which p16INK4a‐positive cells were selectively eliminated. These experiments demonstrated that removal of senescent cells delayed both natural and premature aging in mice, providing proof of concept that targeting cellular senescence can extend healthspan and mitigate age‐associated pathologies (Baker et al. 2011, 2016).

Senolytics induce apoptosis of senescent cells by inhibiting senescent cell anti‐apoptotic pathways. The first senolytic agents identified were dasatinib and quercetin, and since then, a wide range of compounds has been investigated—from natural molecules to kinase inhibitors, HSP90 inhibitors, and p53 pathway modulators, among others (Kirkland and Tchkonia 2020; Xu et al. 2018).

The D + Q combination (dasatinib and quercetin) has even been tested in humans, showing certain effects such as a reduction in senescent cells within adipose tissue and a decrease in circulating SASP factors in the blood (Hickson et al. 2019). In another study involving patients with AD, a reduction in circulating cytokines in the cerebrospinal fluid was also observed (Hickson et al. 2019; Gonzales et al. 2023). However, despite these biochemical improvements, no cognitive enhancement was detected in patients after 12 weeks of treatment. Nevertheless, the initial findings remain promising.

Chang et al. (2016) demonstrated that ABT‐263, a senolytic drug that inhibits anti‐apoptotic proteins of the BCL‐2 family, can promote the clearance of senescent cells, thereby mitigating premature aging of the hematopoietic system and rejuvenating aged hematopoietic and muscle stem cells. In a progeroid mouse model, treatment with ABT‐263 resulted in a 50%–70% reduction of senescent cells across multiple tissues, decreased nuclear factor kappa B (NF‐κB) activation and cytokine expression, and extended lifespan. Treated animals exhibited increased voluntary activity, reduced proportions of senescent cells in organs, lower SASP and hematologic cytokine levels, reduced immune cell infiltration, and less tissue fibrosis. Despite these promising results, the compound also produced adverse effects, including thrombocytopenia and leukopenia, which highlight the need for safer and more selective senolytic agents in future therapeutic applications (Chang et al. 2016).

More recent studies using the same compound demonstrated a reduction in senescent cell burden within bone marrow mesenchymal stem cells, as well as an improvement in bone structure in vivo in a vitamin D deficiency model. However, all studies emphasize that the compound exhibits adverse effects, and that factors such as treatment duration and dosage are critical to its efficacy and safety. Furthermore, not all senescence‐associated parameters are reversed, indicating that while ABT‐263 shows therapeutic potential, its effects remain partial and context‐dependent (Yang et al. 2024).

An 8‐week treatment with quercetin and dasatinib improved learning and spatial memory in aged Wistar rats, accompanied by reduced plasma levels of inflammatory mediators and peripheral SASP components. Treated animals also exhibited changes in dendritic spine architecture in the CA1 region of the hippocampus, supporting a systemic, rather than purely peripheral, effect of senolytic therapy (Krzystyniak et al. 2022). It is worth noting that the study was conducted exclusively in male animals, underscoring the importance of considering sexual dimorphism in future research to better understand potential sex‐specific responses to senolytic therapy.

In a model of vascular cognitive impairment, an intriguing sex‐dependent response was observed after ABT‐263 treatment: treated male mice showed a reduction in the expression of several inflammatory markers and decreased glial cell activation, whereas female mice exhibited the opposite response (Lambert et al. 2025). This finding highlights the importance of investigating sexual dimorphism, as it has already been demonstrated that neuroinflammatory processes differ between males and females, both in magnitude and underlying molecular mechanisms (Mangold et al. 2017).

Collectively, these studies show that targeting cellular senescence holds great therapeutic potential, but its application remains complex and context‐dependent. They demonstrate that while senolytic strategies can mitigate aging‐related damage and improve tissue function, their efficacy and safety depend on multiple factors—including cell type, dosage, treatment duration, and even sex‐specific responses. Together, these findings underscore the need for a refined, personalized, and carefully monitored approach trying to minimize off‐target effects.

In contrast, senomorphics act by blocking signaling pathways involved in SASP expression, such as the p38MAPK, PI3K/Akt, mTOR, and JAK/STAT pathways, as well as transcription factors like NF‐κB (Xu et al. 2015). They can also neutralize the effects of pro‐inflammatory cytokines secreted by the SASP, including IL‐1α, IL‐1β, and IL‐6. Senomorphics may also consist of natural compounds such as rapamycin and resveratrol, or pharmacological agents like metformin, all of which have been shown to modulate senescence‐associated signaling and attenuate the pro‐inflammatory environment without directly inducing senescent cell death (Bharath et al. 2020; Lin et al. 2018).

In the CNS, metformin has been shown to restore hippocampal neurogenesis and improve learning and memory in obese mice by reducing inflammation and modulating microglial activation through the regulation of gut microbiota (Ma et al. 2021). Metformin has also been associated with increased lifespan in several experimental models (Martin‐Montalvo et al. 2013). In a PD model, its action was primarily exerted on astrocytes, where it delayed cellular senescence and contributed to neuronal protection. By directly targeting mitofusin 2—a protein involved in mitochondrial fusion—metformin restored mitochondrial function, which was otherwise impaired, and reduced the release of mitochondrial DNA into the cytosol. This, in turn, downregulated the cGAS–STING pathway, a key mediator of the innate immune response (Wang et al. 2024). In vivo, metformin treatment improved the characteristic behavioral impairments observed in PD mouse models and preserved dopaminergic neurons, further supporting its potential as a neuroprotective senomorphic agent targeting astrocytic and mitochondrial dysfunction.

Another senomorphic compound, rapamycin, acts by inhibiting the mTOR pathway and inducing autophagy, which leads to enhanced synaptic protein levels and improved neuronal plasticity (Gao et al. 2021). Rapamycin has likewise been shown to extend lifespan, further supporting its potential as a therapeutic agent targeting aging‐related mechanisms in the CNS (Harrison et al. 2009). In our study, rapamycin was used as a pharmacological senomorphic agent targeting the mTOR signaling pathway in senescent astrocytes. Inhibition of mTOR restored autophagic and mitophagic flux, reduced the accumulation of dysfunctional mitochondria, and attenuated key molecular and functional markers of astrocytic senescence. Importantly, rapamycin treatment increased astrocyte resistance to mitochondrial stress and partially recovered homeostatic functions, supporting the concept that modulation of mTOR‐dependent pathways can reverse senescence‐associated phenotypes without eliminating astrocytes. These findings position rapamycin as a relevant therapeutic tool to restore astrocyte function and glial homeostasis during brain aging and neurodegeneration (Diniz et al. 2024). Despite these promising effects, the in vivo functional relevance of rapamycin in senescent astrocytes still needs to be validated in the context of the aged brain, where cellular heterogeneity, circuit‐level interactions, and blood–brain barrier dynamics may critically influence therapeutic efficacy.

Senomorphics are particularly appealing in the context of the CNS, as they avoid the elimination of post‐mitotic cells, thereby reducing potential adverse effects. Instead, they act by modulating cellular function, making them suitable for promoting cognition, synaptic plasticity, and overall neuronal resilience without compromising essential neural cell populations (Riessland et al. 2024).

## 8 Targeting Senescent Cells With CAR‐T Cells: A New Frontier in Senotherapy

While senolytic and senomorphic strategies have provided important proof of concept that cellular senescence is a modifiable process, their limited specificity and off‐target effects pose significant challenges, particularly in the context of the CNS. These limitations have driven the search for more precise approaches capable of selectively targeting senescent cells while preserving tissue integrity. In this context, immunotherapeutic strategies based on chimeric antigen receptor T (CAR‐T) cells have emerged as a highly innovative alternative, offering the possibility of cell‐specific recognition and elimination of senescent cells with improved precision and controllability.

The first‐generation CAR‐T cells lacked a co‐stimulatory domain, which limited their activation and persistence. However, modern CAR‐T cells now include co‐stimulatory domains that enable more selective and effective activation. This co‐stimulatory signaling is the key to achieving more precise and less toxic immune responses (Eshhar et al. 1993; van der Stegen et al. 2015).

The application of CAR‐T cells to target cellular senescence is a relatively recent development. In this approach, surface proteins specifically expressed by senescent cells are identified and used as target antigens, allowing CAR‐T cells to mount a directed and specific immune response. This strategy helps to avoid off‐target effects, offering a more precise and controlled means of eliminating senescent cells while preserving healthy tissue (Amor et al. 2020).

Amor et al. demonstrated that the use of immunotherapy is effective in eliminating senescent cells, and that this approach can reverse or attenuate senescence‐associated pathologies such as fibrosis and tissue damage. The application of urokinase‐type plasminogen activator receptor (uPAR)‐CAR‐T cells also showed that adverse effects were acceptable, with no evidence of excessive toxicity (Amor et al. 2020).

Importantly, the research was extended to models of physiological aging, in which a single administration of uPAR‐CAR‐T cells produced long‐lasting preventive effects. Treated mice exhibited improved physical performance and better metabolic function, suggesting that targeting senescent cells through CAR‐T immunotherapy may represent a promising strategy to mitigate the functional decline associated with aging (Amor et al. 2024).

A recent study by Deng et al. (2024) provided the first evidence that CAR‐T cells engineered with a natural killer group 2D receptor (NKG2D) chimeric receptor can selectively eliminate senescent fibroblasts and astrocytes. Stress‐induced ligands such as MICA/MICB and ULBPs are upregulated on the surface of senescent cells, enabling their selective recognition by NKG2D‐based CAR‐T cells, which efficiently eliminate senescent cells through cytotoxic mechanisms while sparing non‐senescent counterparts.

These results pave the way for potential therapeutic applications within the CNS. This finding is particularly promising, as astrocytic senescence has been shown to facilitate and contribute to the development of neurodegenerative diseases, suggesting that targeted immunotherapies could offer new strategies to counteract CNS aging and neurodegeneration (Deng et al. 2024; Ungerleider et al. 2021; Limbad et al. 2020; Han et al. 2024).

The application of CAR‐based cellular therapies to neurodegenerative diseases remains largely unexplored. In both AD and PD, only a limited number of preclinical studies have investigated the feasibility of CAR approaches. In the context of PD, chimeric antigen receptor–engineered macrophages have been employed to selectively recognize aggregated α‐synuclein, promoting the phagocytosis of fibrils and aggregates without inducing neuronal death. Importantly, this strategy was shown to attenuate neuroinflammation, preserve dopaminergic neurons, and improve motor function in in vivo models of PD (Park et al. 2023). In the context of AD, chimeric antigen receptor–engineered regulatory T cells specific for β‐amyloid have been developed as an immune‐modulatory strategy aimed at controlling neuroinflammation, rather than inducing direct cytotoxicity or promoting plaque phagocytosis (Saetzler et al. 2023). Despite their preliminary and early‐stage nature, these studies provide encouraging proof‐of‐concept evidence.

Although senolytic, senomorphic, and CAR‐T‐based therapies have shown promising potential, their current findings should be interpreted with caution. The available evidence remains preliminary, and several adverse effects have been reported. Because these drugs act on signaling pathways shared among multiple cell types, they can produce undesirable off‐target effects (Adkins 2019). This underscores the need to optimize treatment specificity, dosage, and duration, as well as to design strategies that minimize toxicity while preserving therapeutic efficacy.

At present, we are still far from developing an effective and reliable therapy targeting senescent cells, particularly within the CNS. The translational application of these interventions faces significant challenges, including sex dimorphism, which influences both therapeutic response and susceptibility to senescence, and the limitations of experimental models, which make it difficult to extrapolate findings to the human context. When evaluating in vivo effects, it is crucial to consider the complex interplay among different neural cell types and brain regions, the blood–brain barrier, the heterogeneity of the neural microenvironment, and the systemic consequences of these treatments (Liu et al. 2020; Zheng et al. 2025).

A combined therapeutic approach—integrating senomorphic modulation of senescent cells with their selective elimination via senolytics or CAR‐T–based strategies—appears particularly promising. Nevertheless, such approaches require careful refinement to achieve an optimal balance between efficacy and safety.

In summary, while these emerging strategies represent a major step forward in the field of cellular senescence (Table 1), it is essential to advance through rigorous preclinical research that fully accounts for the biological complexity of the CNS. Ultimately, therapeutic efforts should aim to restore and preserve cellular homeostasis without inducing tissue damage or functional impairment.

**TABLE 1:** Therapeutic strategies targeting cellular senescence in the CNS.

Agent	Mechanism of action	CNS evidence	Key references
SENOLYTICS—selective elimination of senescent cells			
Dasatinib + Quercetin (D + Q)	Combined inhibition of multiple SCAPs (Src kinases, BCL‐2 family, PI3K/AKT), increasing senolytic specificity	CNS penetrant; reduced senescent burden, shifted microglia toward homeostasis, improved cognition and synaptic integrity in AD and aging models	(Gonzales et al. 2023) (Hickson et al. 2019) (Xu et al. 2018) (Krzystyniak et al. 2022)
Fisetin	Flavonoid targeting BCL‐xL and pro‐survival pathways, inducing apoptosis in senescent cells	Crosses BBB; reduced Aβ and tau pathology, improved mitochondrial function, extended healthspan in aging and AD models	(Yousefzadeh et al. 2018) (Zhu et al. 2017) (Zhang et al. 2024‐10‐27)
Navitoclax (ABT‐263)	BCL‐2/BCL‐xL inhibitor inducing mitochondrial apoptosis; limited by thrombocytopenia.	Cell‐type–specific CNS senolytic; reduced glial activation in male mice, with sex‐dependent effects and limited brain penetration.	(Zhu et al. 2015); (Lambert et al. 2025) (Chang et al. 2016) (Yang et al. 2024)
SENOMORPHICS—SASP suppression without cell death			
Rapamycin	mTORC1 inhibition suppressing SASP, enhancing autophagy and mitophagy	Improved cognition, reduced astrocyte senescence, restored mitochondrial homeostasis, extended lifespan in aging and PD models	(Gao et al. 2021) (Diniz et al. 2024) (Harrison et al. 2009)
Metformin	AMPK‐dependent and independent suppression of NF‐κB/SASP; improves mitochondrial function	Improved cognition and neurogenesis; delayed astrocyte senescence and preserved dopaminergic neurons in PD models	(Ma et al. 2021; Wang et al. 2024; Bharath et al. 2020)
JAK Inhibitors	Block JAK/STAT signaling to suppress SASP and inflammation	Reduced SASP in aging tissues; CNS applications under investigation	(Xu et al. 2015)
CAR‐T Immunotherapy—engineered T‐cell targeting			
uPAR‐CAR‐T cells	Targets uPAR‐positive senescent cells for cytotoxic elimination	Reduced senescent burden, reversed age‐associated dysfunction, and produced long‐lasting benefits in aging models	(Amor et al. 2020, 2024)
NKG2D‐CAR‐T cells	Targets stress‐induced NKG2D ligands broadly upregulated in senescent cells	Selective elimination of senescent astrocytes and fibroblasts; improved function in aged and irradiated mice; safe in non‐human primates	(Deng et al. 2024; Ungerleider et al. 2021; Yang et al. 2023)

Abbreviations: Aβ, amyloid beta; AD, Alzheimer's disease; AMPK, AMP‐activated protein kinase; BBB, blood–brain barrier; BCL‐2/BCL‐xL, anti‐apoptotic B‐cell lymphoma proteins; CAR‐T, chimeric antigen receptor T cells; CNS, central nervous system; D + Q, dasatinib plus quercetin; JAK, Janus kinase; mTORC1, mechanistic target of rapamycin complex 1; NF‐κB, nuclear factor kappa B; NKG2D, natural killer group 2 member D; PD, Parkinson's disease; PI3K/AKT, phosphoinositide 3‐kinase/protein kinase B; SASP, senescence‐associated secretory phenotype; SCAPs, senescent cell anti‐apoptotic pathways; STAT, signal transducer and activator of transcription; uPAR, urokinase‐type plasminogen activator receptor.

## 9 Concluding Remarks

Aging and neurodegenerative diseases are increasingly recognized as outcomes of progressive cellular dysfunction rather than solely neuronal loss. Throughout this review, we highlight that glial cells, particularly astrocytes, occupy a central position at the intersection of aging, inflammation, metabolic imbalance, and synaptic dysfunction. The accumulation of senescent glial cells and the persistent activation of the senescence‐associated secretory phenotype emerge as key drivers of neuroinflammation, impaired proteostasis, and loss of neural plasticity, ultimately shaping brain vulnerability to neurodegenerative processes.

Advances in biomarker discovery have revealed that senescence can be detected across multiple biological scales, from intracellular hallmarks such as SA‐β‐gal activity, cell cycle arrest, and DNA damage to circulating SASP factors and EVs. These systemic signatures not only provide insights into biological aging but also open new avenues for early diagnosis, disease monitoring, and patient stratification. However, the heterogeneity of senescent phenotypes and their overlap with reactive cellular states underscore the need for integrative, multi‐marker approaches rather than reliance on single indicators.

Therapeutic targeting of cellular senescence represents a promising yet complex frontier. Senolytic and senomorphic agents have demonstrated the feasibility of modulating senescence‐associated damage, but their translation to the clinic is constrained by context dependency, sex‐specific responses, and potential off‐target toxicity. Emerging immunotherapeutic approaches, particularly CAR‐T–based strategies, offer unprecedented specificity and raise the possibility of selectively eliminating senescent cells in a controlled manner. Early experimental evidence suggests that such precision therapies may be especially relevant for targeting senescent astrocytes and other CNS cell populations implicated in neurodegeneration. Nevertheless, eliminating senescent cells must be approached with caution, as senescent glial populations may retain context‐dependent protective or homeostatic functions, and their indiscriminate removal could disrupt essential cellular networks and compromise tissue integrity.

Despite these advances, significant challenges remain. A deeper understanding of senescence heterogeneity, regional vulnerability within the brain, immune surveillance mechanisms, and long‐term consequences of senescent cell removal is essential. Future efforts should integrate rigorous preclinical modeling with longitudinal human studies to define optimal therapeutic windows, dosing strategies, and combinatorial approaches. Ultimately, successful interventions will need to balance the removal or modulation of harmful senescent cells with the preservation of essential cellular functions, aiming not to halt aging per se, but to restore tissue homeostasis, preserve cognitive function, and promote healthy brain aging.

## Author Contributions

**Lívia de Sá Hayashide:** conceptualization, investigation, writing – original draft, writing – review and editing. **Bruna Pessoa:** conceptualization, investigation, writing – original draft. **Gustavo Dias:** investigation, writing – review and editing. **Bruno Pontes:** investigation, writing – review and editing, writing – original draft, supervision. **Rafael Serafim Pinto:** investigation, writing – original draft, writing – review and editing, supervision. **Luan Pereira Diniz:** conceptualization, investigation, writing – original draft, writing – review and editing, project administration, supervision.

## Funding

This work was supported by grants from: Fundação Carlos Chagas Filho de Amparo à Pesquisa do Estado do Rio de Janeiro (FAPERJ), International Society for Neurochemistry (ISN), Conselho Nacional de Desenvolvimento Científico e Tecnológico (CNPq), Instituto de Educação Médica (IDOMED), and Coordenação de Aperfeiçoamento de Pessoal de Nível Superior (CAPES).

## Conflicts of Interest

The authors declare no conflicts of interest.

## Data Availability

Data sharing not applicable to this article as no datasets were generated or analysed during the current study.
